# Medical Data Mining Course Development in Postgraduate Medical Education: Web-Based Survey and Case Study

**DOI:** 10.2196/24027

**Published:** 2021-10-01

**Authors:** Lin Yang, Si Zheng, Xiaowei Xu, Yueping Sun, Xuwen Wang, Jiao Li

**Affiliations:** 1 Institute of Medical Information and Library Chinese Academy of Medical Sciences and Peking Union Medical College Beijing China

**Keywords:** medical data mining, course development, online teaching, postgraduate medical education

## Abstract

**Background:**

Medical postgraduates’ demand for data capabilities is growing, as biomedical research becomes more data driven, integrative, and computational. In the context of the application of big data in health and medicine, the integration of data mining skills into postgraduate medical education becomes important.

**Objective:**

This study aimed to demonstrate the design and implementation of a medical data mining course for medical postgraduates with diverse backgrounds in a medical school.

**Methods:**

We developed a medical data mining course called “Practical Techniques of Medical Data Mining” for postgraduate medical education and taught the course online at Peking Union Medical College (PUMC). To identify the background knowledge, programming skills, and expectations of targeted learners, we conducted a web-based questionnaire survey. After determining the instructional methods to be used in the course, three technical platforms—Rain Classroom, Tencent Meeting, and WeChat—were chosen for online teaching. A medical data mining platform called Medical Data Mining - R Programming Hub (MedHub) was developed for self-learning, which could support the development and comprehensive testing of data mining algorithms. Finally, we carried out a postcourse survey and a case study to demonstrate that our online course could accommodate a diverse group of medical students with a wide range of academic backgrounds and programming experience.

**Results:**

In total, 200 postgraduates from 30 disciplines participated in the precourse survey. Based on the analysis of students’ characteristics and expectations, we designed an optimized course structured into nine logical teaching units (one 4-hour unit per week for 9 weeks). The course covered basic knowledge of R programming, machine learning models, clinical data mining, and omics data mining, among other topics, as well as diversified health care analysis scenarios. Finally, this 9-week course was successfully implemented in an online format from May to July in the spring semester of 2020 at PUMC. A total of 6 faculty members and 317 students participated in the course. Postcourse survey data showed that our course was considered to be very practical (83/83, 100% indicated “very positive” or “positive”), and MedHub received the best feedback, both in function (80/83, 96% chose “satisfied”) and teaching effect (80/83, 96% chose “satisfied”). The case study showed that our course was able to fill the gap between student expectations and learning outcomes.

**Conclusions:**

We developed content for a data mining course, with online instructional methods to accommodate the diversified characteristics of students. Our optimized course could improve the data mining skills of medical students with a wide range of academic backgrounds and programming experience.

## Introduction

Big data holds promise for achieving a new understanding of the mechanisms of health and disease and of making biomedical research more data driven, integrative, and computational. In a survey of 704 National Science Foundation investigators from the Directorate for Biological Sciences [1], 90% reported that they were, or would soon be, analyzing large data sets. Meanwhile, future physicians are actively preparing for this new era of data and digital health. A national survey conducted by Stanford Medicine [2] showed that medical students now pursue supplemental education in data-oriented subjects, such as advanced statistics, coding, and artificial intelligence.

With the aim of training students in data operation and advanced algorithm application via computer programming, data mining courses are designed to develop students’ practical skills in general data structure and program coding [3-6]. For example, “Introduction to Data Mining” at Ohio State University [3] is a project-based course that provides an in-depth understanding of data mining methodology. However, these courses are not specific to health care scenarios. Developing data mining courses that focus on the characteristics of medical data and associated data mining techniques in the context of concrete health care analytic applications is essential for medical postgraduates.

For medical schools, achieving this is more difficult than expected. Firstly, medical data mining courses may attract medical students with diverse academic backgrounds, including public health, oncology, cardiology, neurology, pharmacy, and nursing. Since the role of domain knowledge may be dominant when analyzing data and interpreting results [7], instructors need to be equipped with necessary domain knowledge and programming skills. However, instructors generally lack training or expertise, just as a nationwide survey of US life sciences faculty showed [8]. Secondly, health care analytic applications are diverse, including planning or implementing interventions, disease detection, therapeutic decision support, outcome prediction, and personalized medicine [9]. Different applications vary in scientific problems, data type, analysis paradigms, and techniques. Meanwhile, the medical data are from different sources, involving insurance claims, clinical registries, electronic health records (EHRs), biometric data, patient-reported data, medical imaging, biomarker data, prospective cohort studies, large clinical trials, the internet, and mobile apps [10]. It is still inconclusive as to which type of the above applications should be involved in a practical medical data mining course targeting medical students. Thirdly, learn-to-code courses are largely absent from medical school curricula [11]. Some medical students may have received supplemental education, while others may not have. The diversity of their background knowledge and programming skill level makes course development more difficult, although previous studies showed that medical students who were complete novices at coding were able to create simple, usable clinical programs with 2 days of intensive teaching [12].

To address these problems, some medical schools collaborated with other departments to develop courses, such as the University of Toronto Faculty of Medicine [11]. They developed a 14-month certificate course, “Computing for Medicine,” in collaboration with the Department of Computer Science. Some medical schools developed medical data mining courses focusing on specific data types or specific health care analytic applications. For example, “Collaborative Data Science in Medicine” at the Massachusetts Institute of Technology [13] focused on performing retrospective research using data from EHRs. “Data, Models, and Applications to Healthcare Analytics” at Stanford University delved into applications to medical product safety evaluation and health risk models [14]. Columbia University provides an overview of research methods relevant to biomedical informatics for students in clinical, public health, or translational research programs [15]. Incorporating diversity in a medical data mining course is still a challenging problem.

We aimed to develop an online medical data mining course to accommodate a diverse group of medical students with a wide range of academic backgrounds, programming experience, and motivations. We have an offline course called “Practical Techniques of Medical Data Mining” (No. INSC11011) at Peking Union Medical College (PUMC) [16]. This course started in 2016 and initially targeted medical informatics students with prerequisite course training for computer science. Recently, more and more clinical students have enrolled in our course. Their diversity in programming skills, background knowledge, and needs has brought challenges to our course, which motivates us to incorporate knowledge diversity into our course. To achieve this, previous studies have shown that uncovering potential participants’ needs may be helpful [17-19]. Since medical data mining courses are developed to prepare medical students for data-driven research and the new era of data and digital health, we believe that it is necessary to survey medical postgraduates to identify their perceptions. Accordingly, diversified course content and teaching methods could be designed. For teaching methods, online learning environments offer an opportunity for self-learning and collaborative learning [20,21]. Different web-based platforms have been successfully applied to support different learning processes [22], such as Rain Classroom [23], WeChat [24], DingTalk [25], Zoom [26], Skype, and FaceTime. Compared with traditional face-to-face classes, learning online has advantages in flexibility and virtual communication, and has the potential to deal with the diverse needs of students. Meanwhile, due to the threat of COVID-19, colleges and universities have mandated that faculty move their courses online to help prevent the spread of the virus [27]. In this study, we developed a medical data mining course using internet education technology, aiming to improve the data mining skills of medical students with a wide range of academic backgrounds and programming experience.

## Methods

### Medical Data Mining Course Development Process

#### Overview

**Figure 1:**
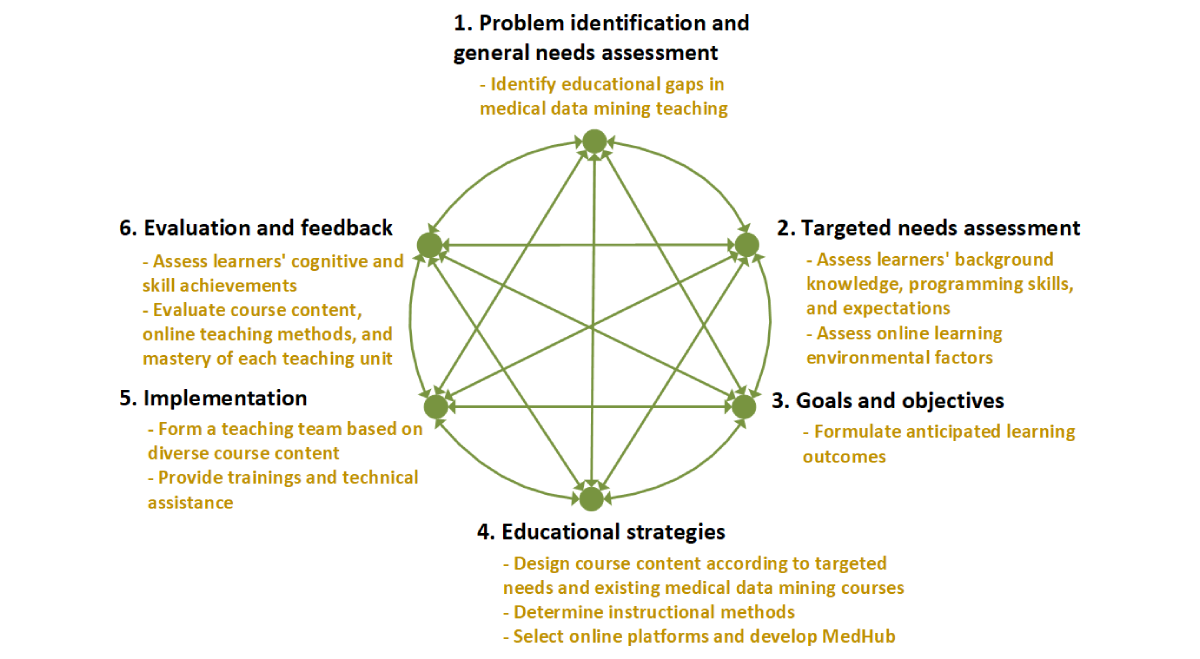
Development process for the medical data mining course. MedHub: Medical Data Mining - R Programming Hub.

The course “Practical Techniques of Medical Data Mining” (No. INSC11011) is offered at PUMC in the spring semester of each academic year, with a cap of 48 students. To optimize both the content and educational format of our online medical data mining course, we utilized a six-step approach [28] to guide its development, as this approach has led to the successful implementation of a variety of traditional and online courses in medicine [29-31]. The step-by-step process is discussed in the following sections (Figure 1).

#### Step 1: Problem Identification and General Needs Assessment

We reviewed published literature, state-of-art medical data mining courses in leading international medical schools, and existing courses at PUMC to identify educational gaps in medical data mining teaching. After evaluating the advantages and disadvantages of traditional face-to-face teaching and online teaching, we clarified how to move the course online.

#### Step 2: Targeted Needs Assessment

To make the course content suitable for targeted learners, we conducted a web-based questionnaire survey among postgraduates of PUMC to understand their diversified characteristics (detailed in the Precourse Survey section), which should be fully considered in the course design. For online learning environments, factors that affected the selection of online teaching platforms, such as local technical support, were evaluated.

#### Step 3: Goals and Objectives

Based on the needs assessment, anticipated learning outcomes were formulated, including the following: (1) mastering medical data mining research design, (2) learning to use data mining tools (ie, R software environment), and (3) mastering skills of medical data processing, analysis, and interpretation.

#### Step 4: Educational Strategies

To facilitate achievement of educational goals and objectives, this step focused on course content design and determination of online instructional methods. For course content design, we first analyzed the demographics of targeted students, evaluated their background knowledge of data science (statistics, R programming, etc), and picked out some expected subtopics for the medical data mining course from the web-based questionnaire. This process allowed us to get some clarity regarding their diversity. Meanwhile, we investigated medical data mining–related courses that are offered in the leading colleges and universities, such as Stanford University, Harvard University, and Columbia University, so that we could note the differences between segments currently taught by these courses and what the students wanted to learn. Based on our abundant investigation and detailed analysis of the requirements, the overall scheme, as well as the targeted content of the course, was then established. We structured the course into eight sessions, with the first three sessions covering the general introduction of medical data mining and R programming, while the following five sessions introduced different medical data mining scenarios that delivered a transformative learning experience that would bring the students to their desired future state.

To meet content objectives and address the diversity of potential target learners, we intended to use a variety of instructional methods. We compared 41 teaching methods [32] to identify the appropriate ones for this course and discussed how to convert them into an online format. After summarizing the advantages and disadvantages of commonly used online teaching technologies and social media platforms (Multimedia Appendix 1), we chose Rain Classroom [33], Tencent Meeting [34], and WeChat [35] for their different instructional methods. Meanwhile, a specific medical data mining platform, named Medical Data Mining - R Programming Hub (MedHub) [36], was developed for self-learning (detailed in the Development of the Medical Data Mining Platform: MedHub section).

#### Step 5: Implementation

The course was open for registration in January 2020 and was online from May to July in the spring semester of 2020. To be qualified for diverse teaching units, our teaching team consisted of 6 investigators from different disciplines, including bioinformatics, medical informatics, statistics, and computer science. To familiarize both learners and participating faculties with online instruction, we, as well as the Graduate School, organized various trainings and provided technical assistance to troubleshoot issues during the course. With the online course, we could track all our students’ progress, figure out how to design our course better, and tweak our teaching style. For example, if we saw that most students performed poorly on a certain chapter quiz, we would review the key points of that chapter and interpret the quiz questions in future iterations of the course.

#### Step 6: Evaluation and Feedback

According to anticipated learning outcomes, we clarified a specific measurable method for learners’ cognitive and skill achievements. Meanwhile, we conducted a postcourse survey (detailed in the Postcourse Survey section) and a case study to validate the effectiveness of our online medical data mining course in benefitting a diverse group of medical postgraduates.

### Web-Based Survey

#### Overview

We conducted pre- and postcourse surveys to understand students’ views on the course. An online survey platform, WJX, was employed to collect survey data, and R (version 4.0.0; The R Foundation) was used for statistical analysis. Survey data were only available to teaching team members for the purpose of course development and assessment. All participants were informed that their responses would be used to inform public-facing research. The ethics committee of the Institute of Medical Information, Chinese Academy of Medical Sciences and PUMC, approved this study (IMICAMS/01/20/HREC).

#### Precourse Survey

The precourse questionnaire consisted of two main parts. The first part comprised a set of demographic questions to capture each participant’s name, student ID, department or faculty, discipline, grade, and email address. The second part consisted of questions to acquire information about the students’ mathematical foundation, programming experience (ie, R and other programming languages), and expectations about the course; expectations were collected in free-text format. Participants were recruited via a WeChat group, which consisted of postgraduates of PUMC who were interested in medical data mining. We collected data at the beginning of the spring semester in 2020 and exported them from the online survey platform to Microsoft Excel 2010. Standard descriptive statistics were used to summarize the data. Qualitative data were analyzed based on human-annotated results.

#### Postcourse Survey

The postcourse questionnaire contained 20 items grouped into three topics: course content assessment, online teaching methods assessment, and mastery of each teaching unit. Items that assessed attitudes toward course content included reasonable knowledge structure, front-edged teaching content, the content’s integration with clinical practice and research, and practicality. The evaluation of online teaching methods mainly focused on the functions and teaching effects of four platforms: Rain Classroom, Tencent Meeting, WeChat, and MedHub. Responses to items under the topics “course content assessment” and “mastery of each teaching unit” were recorded on a scale that ranged from 1 (“very negative”) to 5 (“very positive”). For the topic “online teaching methods assessment,” we chose a 3-point scale with the options “dissatisfied” (score=1), “neutral” (score=2), and “satisfied” (score=3). Enrolled students and auditors were invited to participate in the survey. Data collection took place in July 2020.

### Development of the Medical Data Mining Platform: MedHub

**Figure 2:**
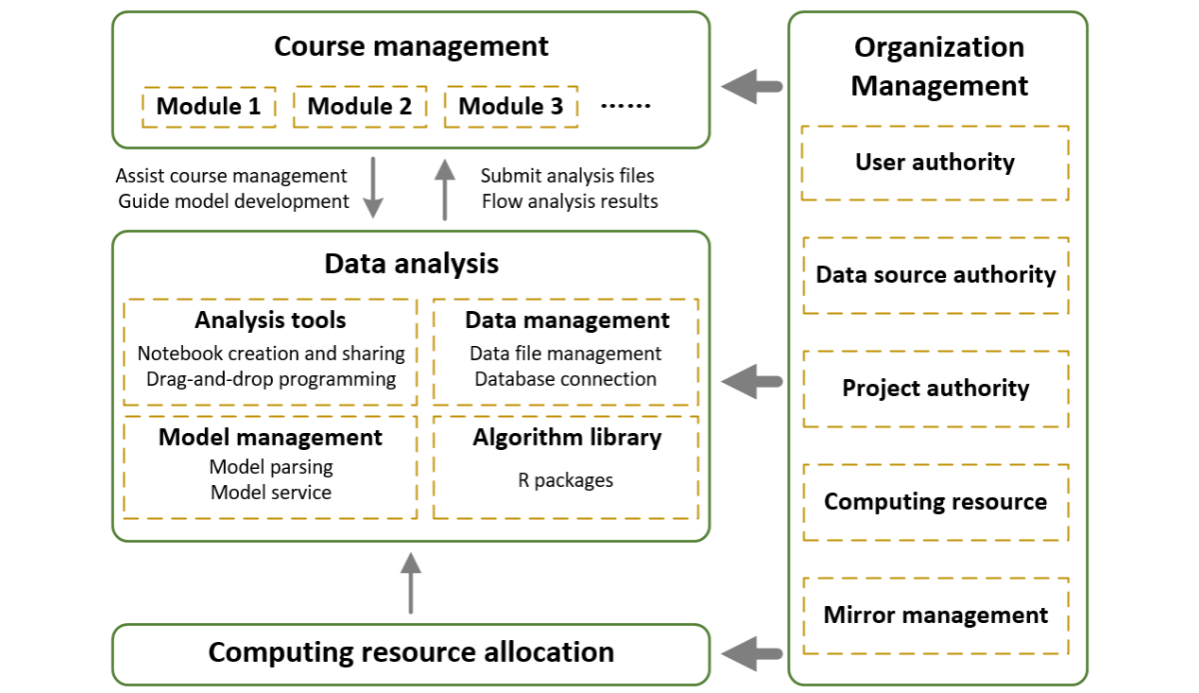
MedHub (Medical Data Mining - R Programming Hub) system architecture.

We designed and developed MedHub [36] based on the Jupyter Notebook [37], aiming to help students build their computational thinking and avoid engineering troubles in the process of coding and environment configuration. Considering their limited available time [38]—especially under the influence of COVID-19 [39]—and diverse expectations, it was necessary to integrate multimedia learning materials into the platform to facilitate on-demand self-learning. As a result, MedHub consisted of four modules (Figure 2), as follows:

1. Computing resource allocation module. A Kubernetes-based [40] computing resource allocation module was used for automating deployment, scaling, and management of containerized applications. It could provide an online R programming workspace for authorized users. Once medical data mining tasks were performed, the customized workspace could allocate computing resources and produce the results.
2. Data analysis module. This module contained core functions required for medical data mining, including analysis tools, data management, model management, and algorithm library. It allowed authorized users to upload data sets, import R packages, and execute medical data mining tasks.
3. Course management module. This module was used to create courses, add course content (ie, multimedia files, data sets, projects, and homework), create and edit notebook courseware (eg, R markdown files), and manage homework.
4. Organization management module. This module was employed to help system administrators manage students, instructors, and groups; clarify access rights; assign different computing resources to different groups; and manage the mirror environment.

Website security was guaranteed through an authentication mechanism with usernames and passwords.

## Results

### Diversified Characteristics of Target Learners

A total of 200 medical postgraduates from 30 disciplines at PUMC participated in the precourse survey (Table 1). Most of them (n=137, 68.5%) were clinical students, majoring in internal medicine (n=57, 28.5%) and surgery (n=28, 14.0%), among other fields. The survey results showed that future physicians were indeed preparing for the era of data and digital health. Although 73.7% of clinical students (101/137) had no programming experience, the majority (108/137, 78.8%) had background knowledge of statistics, which could help them to understand data mining methods. Since 75.5% (151/200) of potential target learners had knowledge of statistics, we could pay less attention to mathematical fundamentals in the course. Compared with other disciplines, public health and preventive medicine students had a good foundation in statistics (17/17, 100%) and programming (12/17, 71%). For biology students, half of them had basic programming skills in R, while over half had knowledge of statistics (12/22, 55%). Meanwhile, survey data showed that students with programming experience were distributed across different disciplines. A total of 63% (45/72) had the ability to code in R, and 31% (22/72) knew at least two programming languages (eg, R and Python). Such distribution drove us to comprehensively consider the explanation of basic programming and the theoretical basis of data mining when designing course content. Meanwhile, the diversity of programming skills and data science background knowledge indicated the feasibility of collaborative learning.

We further analyzed the survey data available in textual format and summarized the participants’ expectations for the medical data mining course (Table 2). It was worth noting that each participant might propose various concerns about the course. Survey data showed that the majority of respondents (68/109, 62.4%) were taking the course for research purposes. They expected to make use of omics data (33/109, 30.3%) and EHRs (20/109, 18.3%). Numerous open data sets were mentioned, including the Medical Information Mart for Intensive Care III [41], the Gene Expression Omnibus [42], and The Cancer Genome Atlas (TCGA) [43]. For clinical data, they realized the importance of information extraction and data cleaning and expected to master relevant skills. For omics data, they mainly wanted to learn how to analyze an open data set from start to finish. As 64.0% of participants (128/200) had no programming experience, 51 (25.5%) expressed their desire to develop practical skills in program coding. Their main concern was learning to use R programming languages to complete specific analysis tasks (33/109, 30.3%) and visualize results (11/109, 10.1%). Moreover, 35.8% of respondents (39/109) expected to understand the data mining methodology, especially machine learning (16/109, 14.7%). A total of 11.9% (13/109) of participants needed to build computational thinking, which would help them design data mining studies.

**Table 1:** Demographics of participants.

Demographics				Participants (N=200), n (%)
**Academic discipline**				
	**Clinical medicine**			
		Total	137 (68.5)	
		Internal medicine	57 (28.5)	
		Surgery	28 (14.0)	
		Oncology	13 (6.5)	
		Obstetrics and gynecology	10 (5.0)	
		Imaging medicine and nuclear medicine	7 (3.5)	
		Others	22 (11.0)	
	**Biology**			
		Total	22 (11.0)	
		Biochemistry and molecular biology	17 (8.5)	
		Others	5 (2.5)	
	**Public health and preventive medicine**			
		Total	17 (8.5)	
		Epidemiology and health statistics	15 (7.5)	
		Others	2 (1.0)	
	**Basic medicine**			
		Total	9 (4.5)	
		Medical informatics	3 (1.5)	
		Stem cells and regenerative medicine	2 (1.0)	
		Others	4 (2.0)	
	**Pharmaceutical science**			
		Total	7 (3.5)	
		Pharmacology	3 (1.5)	
		Others	4 (2.0)	
	Library, information, and archival sciences			6 (3.0)
	Public management science			2 (1.0)
**Training program**				
	Doctor of Medicine or Doctor of Philosophy			98 (49.0)
	Master’s program			102 (51.0)
**Background knowledge of statistics**				
	Yes			151 (75.5)
	No			49 (24.5)
**Programming experience**				
	No programming experience			128 (64.0)
	Only R			23 (11.5)
	Only other programming languages			27 (13.5)
	R and other programming languages			22 (11)

**Table 2:** Participants’ expectations about the course.

Expectations of the course				Participants (N=200), n (%)		Examples of typical statements^a^
**Research**						
	Total participants taking the course for research purposes			68 (34.0)		N/A^b^
	**Expectations^c^**					
		Omics data analysis	33 (16.5)		我想学习基因的差异性表达分析. (I want to learn differential gene expression analysis.)	
		Clinical data analysis	20 (10.0)		希望学习如何从病历中提取数据进行研究. (I want to know how to extract and mine electronic medical record data.)	
		Text mining	2 (1.0)		对文本挖掘比较感兴趣. (I am interested in text processing.)	
		Others	18 (9.0)		希望能讲一下图像的影像组学，特别是神经影像. (I expect the course will include radiomics, especially neuroimaging.)	
**Programming**						
	Total participants taking the course to learn about programming			51 (25.5)		N/A
	**Expectations^c^**					
		R	33 (16.5)		期待应用R语言实现聚类分析等生信分析. (I look forward to using R to perform bioinformatics analysis such as cluster analysis.)	
		Draw function	11 (5.5)		希望会做火山图、热图、气泡图等. (I want to know how to generate volcano maps, heat maps, bubble maps, etc.)	
		General	11 (5.5)		希望代码示例能够有详细讲解或注释. (I would like the codes to be explained or commented on in detail.)	
		Others	2 (1.0)		熟悉常用医学统计软件使用. (I expect the course will help me get familiar with statistical software.)	
**Data analysis and mining methods**						
	Total participants taking the course to learn about data analysis and mining methods			39 (19.5)		N/A
	**Expectations^c^**					
		Machine learning	16 (8.0)		学习用临床数据制作手术前危险因素对术后预后的预测的临床预测模型. (I expect to learn how to use clinical data to establish a predictive model of preoperative risk factors for postoperative prognosis.)	
		Computational thinking	13 (6.5)		希望能够掌握数据挖掘的基本思路和方法. (I expect to master the basic ideas and methods of data mining.)	
		General	10 (5.0)		希望学会文献里常用的一些数据分析方法. (I expect to learn data analysis methods commonly used in scientific literature.)	
		Statistical analysis	7 (3.5)		学习不同的统计建模方法的原理和应用场景. (I expect to learn the principles and application scenarios of different statistical modeling methods.)	
		Deep learning	3 (1.5)		对深度学习有一定理解，初步进行分析. (I want to learn deep learning and be able to perform preliminary data analysis.)	
Other expectations				11 (5.5)		希望能跟着老师做几个实际的案例. (I want to follow the teacher to do some cases.)
No expectations				91 (45.5)		N/A

^a^Example statements are reported in Chinese, followed by their English translations.

^b^N/A: not applicable; statements were provided only for specific expectations.

^c^Participants could have multiple expectations about the course.

### Course Content Design Toward Improving Data Mining Practical Skills of Medical Postgraduates

According to the surveyed programming skills, experiences, background knowledge, and learning expectations of targeted students, we designed the course so that it focused on the combination of theory and practices to achieve good teaching outcomes. The designed content covered the theoretical introduction of expected subtopics as well as the relevant medical data mining cases and practical analyzing strategies. In this way, students could be highly engaged and could practice throughout the class. Generally, according to the curriculum arrangement, we structured the course into eight logical teaching units or sessions, each comprised of four theoretical lessons and two practical lessons. Considering that 64.0% (128/200) of learners had no programming experience, the first three sessions covered the general introduction of medical data mining and R programming. This would lay a foundation for the study and practice topics that would follow. Summarization of the participants’ expectations for the medical data mining course showed that omics and clinical data analysis were hot domains in research, and the literature supports this [44-47]. In addition, many students were interested in the methods used for data analysis and data mining. Therefore, we designed five different medical data mining subtopics to be included in Sessions 4 to 8, to introduce commonly used data cleaning strategies, machine learning models, clinical text mining, gene expression analysis, and the transformation of medical data mining into online application tools (Table 3). Each session would summarize the basic research methods and the recent progress in the theoretical portion, while the practice lesson would demonstrate how to complete a specific health care analytic application from start to finish using R. To accommodate diverse medical students, we included diversified health care analysis scenarios (eg, gene expression analysis and clinical named entity recognition [CNER]), diversified data types (eg, omics data and EHRs), and associated data mining techniques (eg, using R packages to perform CNER based on conditional random fields [CRFs]).

The last session would be the final exam. Based on anticipated learning outcomes and student perceptions, we designed a three-step method to assess student achievement:

1. Problem-solving case study (30% of their final mark). Students needed to apply R to solve practical problems, such as handling outliers in a specified data set.
2. Reading report (30% of their final mark). Students needed to write reading reports to show how much information they understood and grasped from a medical data mining paper.
3. Group project (40% of their final mark). Students were divided into groups. Each group collaborated to complete a complex data mining project and gave an oral presentation.

Faculty members involved in the course would give a comprehensive score based on students’ performance in these three aspects.

**Table 3:** Optimized course content.

Week and module	Teaching content
Week 1. Introduction to medical data mining	Conceptual introduction to medical data mining, as well as the ideas behind turning data into actionable knowledge. Practical introduction to tools (R and RStudio) that will be used in the program.
Week 2. R programming (1)	Install and configure software necessary for programming environment. Introduction to R basic programming, including accessing R packages, import data with R, R functions, and data visualization. Examples for profiling R code.
Week 3. R programming (2)	Descriptive and exploratory data analysis with R (t test, regression models, generalized linear models, etc) and R markdown. Examples for profiling R code.
Week 4. Data acquisition and cleaning	Data interface with R, which will cover the basic ways that data can be obtained. Data cleaning with R (missing values, outliers, error data, and inconsistent data). Examples for profiling R code.
Week 5. Machine learning models for medical data	Introduction to a range of machine learning models, as well as the process of building and applying prediction functions with emphasis on practical applications with R programming. Examples for profiling R code.
Week 6. Clinical text mining	Conceptual introduction to text mining. Summarization of methods and workflow for medical text mining. Case study: clinical named entity recognition for electronic health records.
Week 7. Data mining for biomarker discovery	Introduction to computer-aided biomarker discovery. Regular pipeline for gene expression analysis with R. Case study: differential gene expression analysis.
Week 8. Development of medical data mining tools	Introduction to interactive web application construction, including the basics of creating data products using Shiny, R packages, and interactive graphics. Case study: development of medical data mining tools.
Week 9. Exam and final presentation	Assessment method: oral presentation of group projects; the primary measure is the understanding and knowledge of tools and ideas for medical data mining.

### Teaching Strategies Using Internet Technology

#### Instructional Methods for Online Teaching

To meet content objectives and various expectations, we adopted eight instructional methods for our online course (Table 4). Demonstration, problem solving, and a group project were core methods of skill education [48], while self-learning was a modern method emerging with the rise of the internet. Note that students in collaborative learning groups should be as diverse or heterogeneous as possible. In this way, students with different background knowledge or skills could strengthen their existing skills by teaching others and, in turn, learn new skills from other group members.

Accordingly, we selected three online platforms and developed MedHub to convert instructional methods into an online format (Table 4). Roughly, Rain Classroom, Tencent Meeting, and WeChat were used for theoretical lectures, live demonstrations, and discussion, respectively, while MedHub was used for self-learning (detailed in MedHub for Self-learning section) and homework submission. For the case study, the instructor would use Rain Classroom to present case content and establish a framework for analysis and would then use Tencent Meeting to lead students to solve the case in real time.

Since each module consisted of independent activities, discussions, required reading, individual or group tasks, and flexible use of various platforms, students could learn on demand or by preference.

**Table 4:** Online platforms corresponding to instructional methods.

Instructional method	Online platform			
	Rain Classroom	Tencent Meeting	WeChat	MedHub^a^
Lecture	✓^b^			
Demonstration		✓		
Discussion			✓	
Case study	✓	✓		✓
Problem solving				✓
Self-learning	✓			✓
Reading report				✓
Group project		✓	✓	

^a^MedHub: Medical Data Mining - R Programming Hub.

^b^Check marks signify that the indicated platforms were used for the indicated methods.

#### MedHub for Self-learning

**Figure 3:**
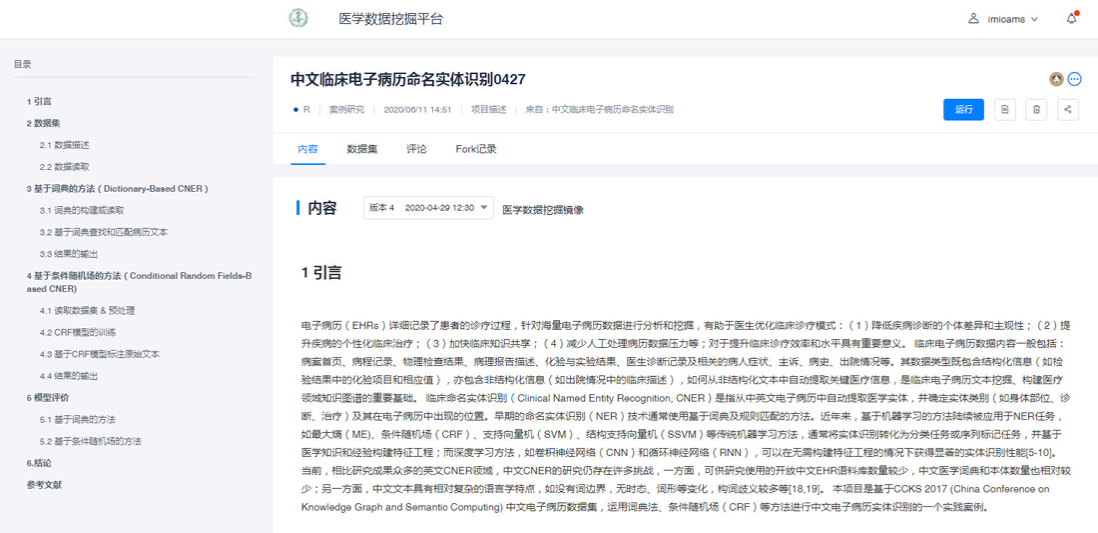
Example of case study in MedHub (Medical Data Mining - R Programming Hub).

MedHub, a web-based application, allowed students to learn by themselves on demand. To achieve this, instructors needed to organize multimedia learning materials for each teaching unit (PowerPoint courseware, data sets, codes, videos, papers, websites, etc) in a structured manner. For case studies, they could share R markdown files containing live code, equations, graphics, visualizations, and narrative text. Experimental data might be provided in a separate file (eg, a comma-separated values file) or be imported programmatically; for instance, by including code in the notebook to download the data from a public internet repository. For the computing environment, system administrators configured the platform with the R environment (version 3.6.0; The R Foundation) as well as packages commonly used in biomedical data mining. Meanwhile, they grouped students according to their characteristics and clarified their access rights to different resources. Authorized students could access various learning materials. Since MedHub provided an online programming workspace with a customized environment, students could create a copy of the R markdown file and run code segments via a web browser (Figure 3). They could also write code based on their own data. Once the code was executed, the platform would allocate computing resources and produce the results so that students could learn how the code worked line by line, with live feedback along the way.

### Practicable Implementation and Demonstration

In 2020, the outbreak of COVID-19 disrupted normal teaching and studying in the field of medical education. To ensure the orderly progress of teaching work, online teaching was put forward by the Graduate School of PUMC. This 9-week course was online from May to July in the spring semester of 2020, with one module per week (roughly 4 hours of student engagement time per week). A total of 6 faculty members and 317 students participated in the course, of which 48 were enrolled students and 269 were auditors.

**Figure 4:**
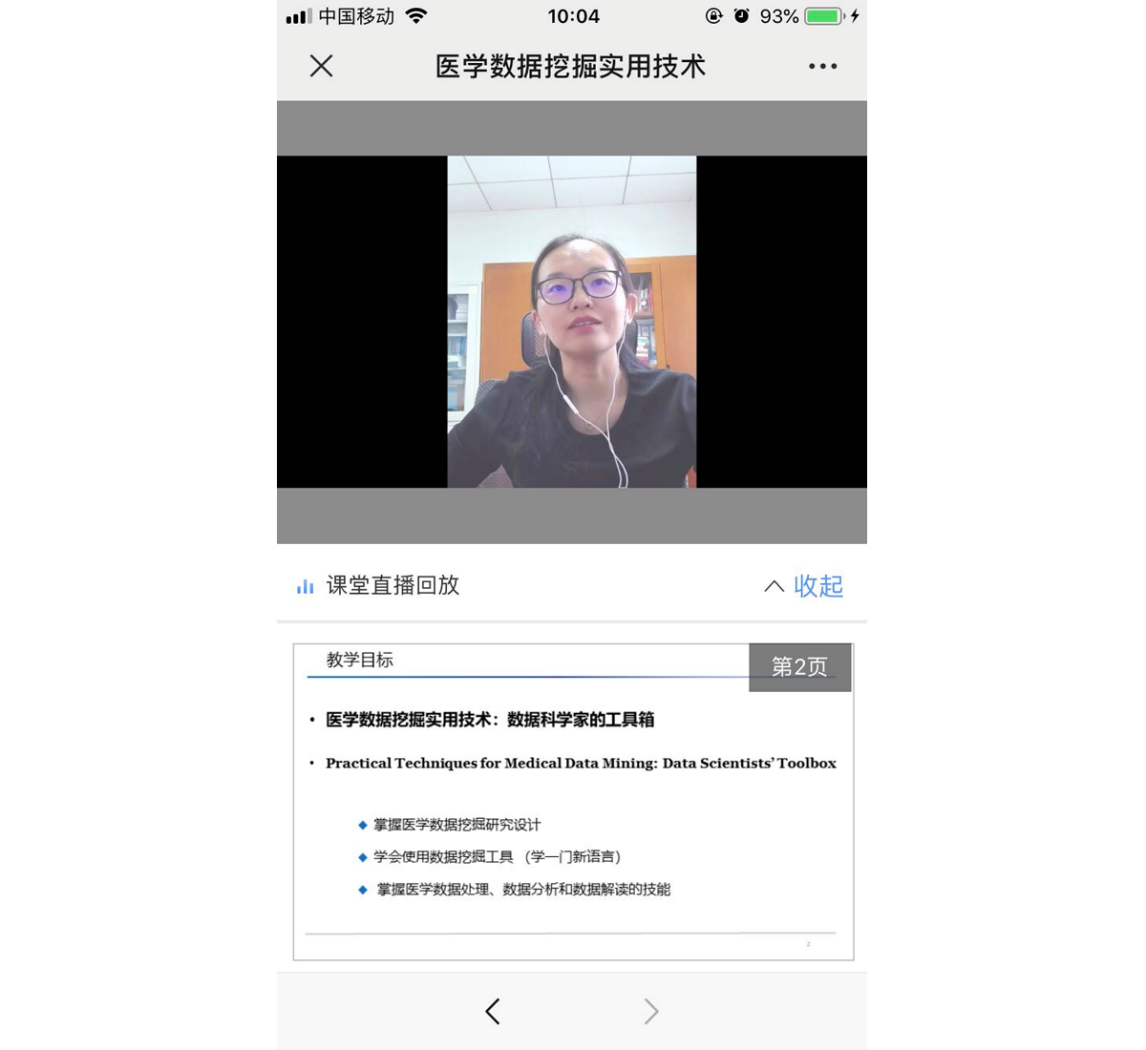
Screenshot of a theoretical lecture in Rain Classroom.

The practicable implementation of the medical data mining course contained the following aspects. For the theoretical teaching portion, educational resources (PowerPoint courseware, data sets, codes, videos, etc) were provided to students in advance for prelearning through Rain Classroom, the WeChat group, and MedHub. During the class, the instructor conducted theoretical lectures by entering Rain Classroom from the PowerPoint slideshow. Once students used WeChat to scan a QR (Quick Response) code to enter the Rain Classroom, the PowerPoint courseware was synchronized with their mobile phones (Figure 4). Students internalized knowledge under the guidance of the instructor, and they gave feedback using multiple interactive methods, such as bullet-point screen comments and the “do not understand” button.

**Figure 5:**
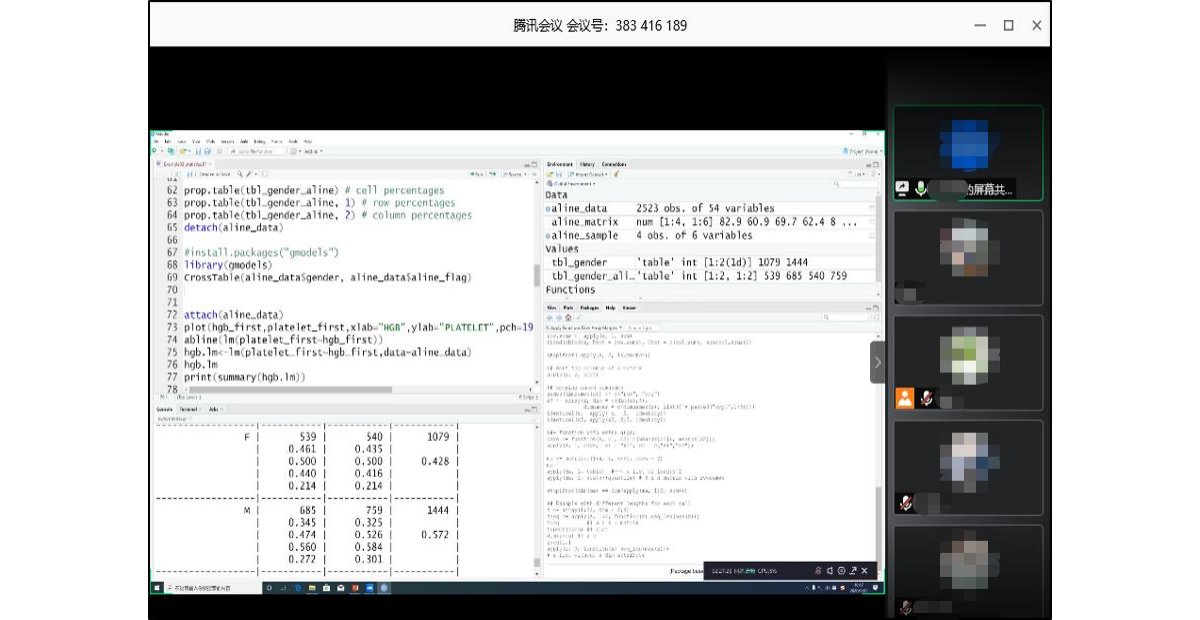
Screenshot of a live demonstration using RStudio in Tencent Meeting.

For the practical teaching portion, instructors used Tencent Meeting to demonstrate how to perform data analysis operations in RStudio (Figure 5). After joining the video conference via their mobile phones, students followed the instructor to complete relevant operations synchronously on their own computers. To practice complex cases such as CNER, which comprised data preparation, dictionary-based CNER, CRF-based CNER, and evaluation, MedHub was used instead of RStudio. When programming errors occurred, students preferred to send instant messages or upload screenshots in the WeChat group for help. Other faculty members and students would give solutions based on their experience.

**Figure 6:**
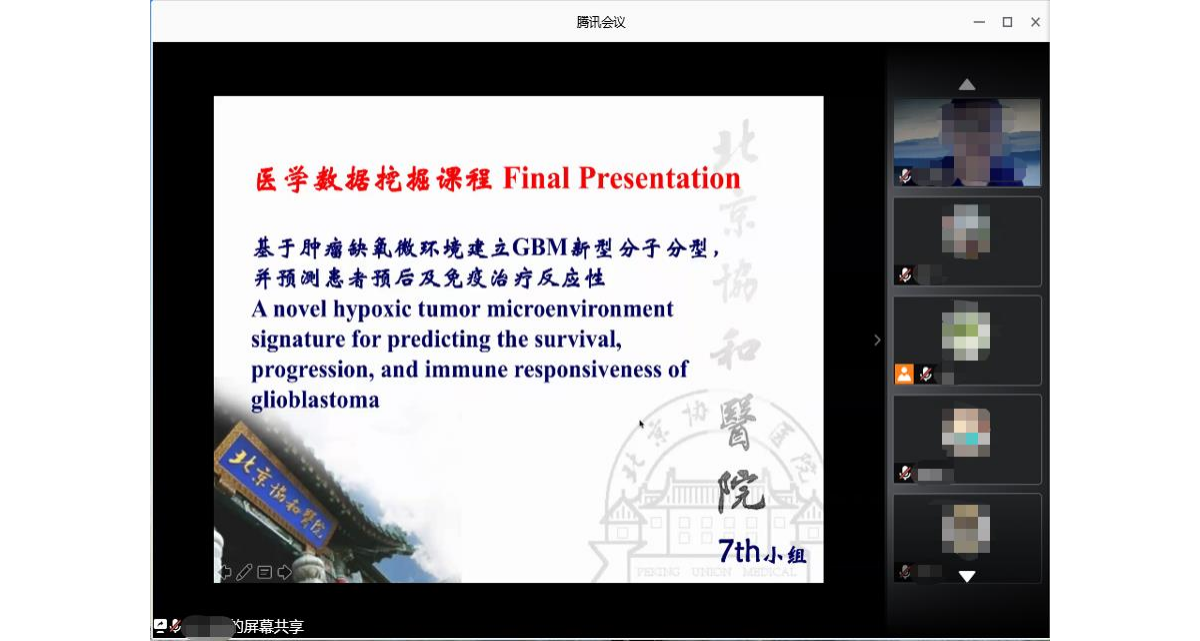
Screenshot of a group presentation in Tencent Meeting.

After the class, students could review what they had learned, and they completed individual and group tasks (ie, problem-solving case studies, reading reports, and group projects) that were released by the instructor to reinforce their skills. For the final group project after 1 month, 48 enrolled students were divided into eight groups. Each group had a leader who was responsible for organizing group members to discuss and complete the group project, as well as a tutor who aimed to give guidance. Group members selected a project from a given list, designed their research, and used R to perform data cleaning, modeling, and visualization, among other tasks. Finally, an oral presentation was given to show the whole process (Figure 6). All of the faculty members assessed each group member according to their performance in the project as well as in the question-and-answer session.

### Course Assessment

To validate the effectiveness of our online medical data mining course in benefitting medical postgraduates with diverse backgrounds, we conducted a postcourse survey with a total of 83 participating students (Table 5). Survey data showed that they found our course to be very practical (n=83, 100% indicated “very positive” or “positive”). A total of 82% (n=68) of the students stated that the content integrated highly with their clinical or scientific problems. For online learning methods, MedHub received the best feedback, both in function (n=80, 96% chose “satisfied”) and teaching effect (n=80, 96% chose “satisfied”), while Rain Classroom performed poorly in function (n=63, 76% chose “satisfied”). In addition, participants’ mastery of the content gradually decreased with the progress of the course: average self-rating scores ranged from 4.78 (SD 0.44) to 4.00 (SD 1.02). Starting from Module 5, several students were unable to keep up with the course at all (n=2, 2% chose “very negative”). Even so, the majority of respondents could master relevant knowledge and skills for each module: responses of “very positive” or “positive” were given by 82 (99%) respondents for Module 1; 78 (94%) respondents for Module 2; 75 (90%) respondents for Module 3; 70 (84%) respondents for Module 4; 64 (77%) respondents for Module 5; 61 (73%) respondents for Module 6; 65 (78%) respondents for Module 7; and 57 (69%) respondents for Module 8.

**Table 5:** Participants’ feedback regarding the course (n=83).

Feedback item					Score, mean (SD)			
**Course content^a^**								
	Reasonable knowledge structure			4.86 (0.39)				
	Front-edged teaching content			4.82 (0.39)				
	Good integration with clinical practice and research			4.81 (0.43)				
	Practicality			4.89 (0.31)				
**Online teaching methods^b^**								
	**Rain Classroom**							
		Platform function	2.73 (0.50)					
		Teaching effect	2.89 (0.35)					
	**Tencent Meeting**							
		Platform function	2.86 (0.35)					
		Teaching effect	2.90 (0.30)					
	**MedHub^c^**							
		Platform function	2.96 (0.19)					
		Teaching effect	2.96 (0.19)					
	**WeChat**							
		Platform function	2.94 (0.24)					
		Teaching effect	2.93 (0.26)					
**Mastery of each module^a^**								
	Module 1. Introduction to medical data mining			4.78 (0.44)				
	Module 2. R programming (1)			4.60 (0.68)				
	Module 3. R programming (2)			4.46 (0.80)				
	Module 4. Data acquisition and cleaning			4.37 (0.81)				
	Module 5. Machine learning models for medical data			4.16 (0.96)				
	Module 6. Clinical text mining			4.10 (0.97)				
	Module 7. Data mining for biomarker discovery			4.14 (0.96)				
	Module 8. Development of medical data mining tools			4.00 (1.02)				

^a^Responses to items under this topic were recorded on a scale ranging from 1 (“very negative”) to 5 (“very positive”).

^b^Responses to items under this topic were recorded on a scale ranging from 1 (“dissatisfied”) to 3 (“satisfied”).

^c^MedHub: Medical Data Mining - R Programming Hub.

## Discussion

### Principal Findings

The growing demand for data mining skills among medical postgraduates prompted us to develop an online medical data mining course at PUMC, exploring how to improve the data mining skills of medical students with a wide range of academic backgrounds and programming experience. According to a six-step approach for course development, combining student expectations and new internet technologies, the course was successfully launched in the spring semester of 2020. Once online, it attracted wide attention, and a total of 317 students participated in the course. Postcourse survey data showed that our course was very practical (n=83, 100% indicated “very positive” or “positive”), and MedHub received the best feedback, both in function (n=80, 96% chose “satisfied”) and teaching effect (n=80, 96% chose “satisfied”).

Our course design was learner centered. To understand who our students were, we used a precourse questionnaire survey to get some clarity regarding their academic backgrounds and programming experience (Table 1). Survey data showed that 68.5% (137/200) of respondents were clinical students, which was consistent with the findings from Stanford Medicine [2]. However, 73.7% (101/137) of students had no programming experience, even though the majority (108/137, 78.8%) had background knowledge of statistics. The data revealed that the lack of necessary background knowledge and skills was the main obstacle to medical data mining education, which has also been verified in bioinformatics education [8]. To minimize the prerequisites, we introduced the basic knowledge and skills of R programming at the beginning of the course, aiming to lay a foundation for the study and practice topics that would follow. According to the responses, 94% (78/83) and 90% (75/83) of the participants were able to master relevant knowledge and skills in Modules 2 and 3, respectively.

The precourse survey was also used to collect, analyze, and interpret the diverse concerns and expectations of our potential learners (Table 2). The survey results showed that the majority of students (68/109, 62.4%) took the course for research purposes and many expected to make use of omics data (33/109, 30.3%) and EHRs (20/109, 18.3%). Thereby, the optimized course covered data acquisition and cleaning, machine learning modeling, clinical data mining, omics data mining, and other content that the students cared about. Simultaneously, a team of teachers with multidisciplinary backgrounds were equipped to teach the course content. To help students translate theoretical knowledge into necessary data mining skills, we developed some representative and typical programming examples or case studies—including “predicting mortality of ICU (intensive care unit) patients,” “differential gene expression analysis,” and “CNER”—for each module, which could assist the students in gaining a rapid understanding of the problem-solving process. These case studies were also chosen to ensure that a variety of techniques were available as useful tools to help students answer the questions. Data mining tasks based on open accessible data sets were introduced in our course as data mining case studies. Strictly following the data access permission, we used the demo codes in the textbook [49] and task publications [50-53] and required the students to apply for data use permissions according to their corresponding licenses. Responses from students indicated that the content integrated highly with their clinical or scientific problems (68/83, 82% chose “very positive”) and that the knowledge structure was highly reasonable (72/83, 87% chose “very positive”). Meanwhile, 77% (64/83) of the students pointed out that the case studies were very helpful for understanding medical data mining knowledge and skills. This indicated that our learner-centered approach was effective for skill-based education, which has been validated by existing research on competency-based education [54,55]. Nevertheless, some content might be difficult for students with weak foundations (2/83, 2% chose “very negative”). In the future, we will design the complex sessions (ie, Sessions 4 to 8) with scenarios, which will be divided into step-by-step and operable units from medical data processing to machine learning model installation. Thus, it will help the students understand the content.

To convert the offline course to an online format, various kinds of online platforms, such as Rain Classroom, Tencent Meeting, WeChat, and MedHub, were used for different instructional methods (Table 4). Relationships between classroom teaching, online teaching, and students’ self-learning are established through mobile phones to achieve long-term efficiency in teaching. Through course implementation, we found that the online format attracted more students to participate in the course. We started by creating a WeChat group that involved 48 enrolled students in order to facilitate communication. Later, more students joined the group via invitations from their classmates. The number of group members exceeded 300 within a few days, which increased the diversity of the students. According to our observations, students with a good foundation in programming and background knowledge were active in the online environment. They were willing to share learning materials and help others, which enabled us to achieve good results in group projects. Eight groups were able to flexibly use knowledge and skills learned to solve various clinical and scientific research problems based on diverse data sets. Some even used algorithms, models, and R packages that were not included in the course.

MedHub, a medical data mining platform, performed impressively as part of our online course. It received the best feedback, both in function (80/83, 96% chose “satisfied”) and teaching effect (80/83, 96% chose “satisfied”). Among all its functions, shared R markdown files containing live code, visualizations, and narrative text were considered the most helpful for authorized students (67/83, 81%), followed by one-stop navigation and downloading of learning materials (61/83, 73%). Students with a poor foundation in programming reported that it was difficult to keep up with instructors to complete operations synchronously on their own computers. The abundant learning resources and demonstration of case studies on MedHub enabled students to review what they had learned and to avoid omissions after class, especially for content that was hard to understand and master. For those with a good foundation in programming and background knowledge, providing more advanced knowledge and skills was important. The online programming workspace with customized environment on MedHub helped them to explore their own data sets, and the extended reading materials allowed them to expand their knowledge. Compared with other biomedical data mining platforms (eg, DrBioRight [56]), our web-based application had an educational purpose, aiming to accommodate a diverse group of medical students.

To validate the effectiveness of our online medical data mining course in helping to improve the data mining skills of medical students with diversified academic backgrounds and programming experience, we randomly selected one group to conduct a case study. Out of 6 group members, 4 (67%) participated in the pre- and postcourse survey; they were majoring in internal medicine, surgery, oncology, and information science. The group leader had no programming experience. From this course, he expected to learn R and analytic applications related to clinical and basic medicine. Through our 9-week course, he was able to lead the group to complete a project—“基于数据挖掘的胃癌微环境及单基因分析” (“Microenvironment and Single Gene Analysis of Gastric Cancer Based on Data Mining”)—by applying the data mining workflow he designed. In addition, he was able to use R to perform microenvironment analysis and visualize the results. The group member who was majoring in oncology had neither programming experience nor statistical knowledge. The precourse survey results showed that she expected the course to teach her how to mine TCGA data. After the course, she was able to use R and Perl to integrate the clinical and transcriptome data of gastric cancer patients from TCGA into a matrix, so that other group members could perform microenvironment analysis and single gene analysis. The group member who was majoring in internal medicine had basic programming skills in R and wanted to learn more advanced data mining techniques. His feedback showed that vivid health care analysis cases in the course made obscure machine learning algorithms easy to understand. He had been able to apply the knowledge and techniques learned to solve his own data mining tasks and had obtained extended learning materials for further study. The group member who had the ability to code in other programming languages expressed his desire to master R. According to his postcourse self-evaluation, he was able to master relevant knowledge and skills from each module (five modules were rated as “very positive,” while others were rated as “positive”). In addition, he was able to collaborate with other group members to complete the analysis of a data set from start to finish using R. This case study showed that our course was able to fill the gap between students’ expectations and learning outcomes, regardless of their academic backgrounds, programming experience, and motivations.

To prepare medical students for data-driven research and the new era of data and digital health, it would be ideal for medical schools to provide a series of medical data mining courses for diverse medical students. Considering that achieving this is currently difficult for most medical schools, incorporating diversity into course content and teaching methods in a medical data mining course has become important. Previous studies have demonstrated diversified course content and teaching methods in neuroscience and nursing [57,58]. However, medical data mining courses still lack exploration. Thereby, we demonstrated how to incorporate diversity into a medical data mining course in a medical school. Our experience showed that designing course content and online instructional methods that accommodated the diversified characteristics of medical students was an effective method of course development. The results showed that our course was able to fill the gap between student expectations and learning outcomes. This process could be helpful to course designers in similar situations.

### Limitations

Our study has two limitations. First, we did not compare learners’ data mining skill levels before and after the class to validate the effectiveness of our online course in improving data mining skills. Instead, we used the self-evaluation of learners in a postcourse survey and a case study, which might make the results somewhat subjective. We will conduct more rigorous validation in the future.

Second, our online course has not yet been accredited by an external organization. After this pilot study has demonstrated the feasibility of the medical data mining course at PUMC, we will apply for a training program from the Chinese Medical Association [59] and the Chinese Society of Academic Degrees and Graduate Education [60].

### Conclusions

In this study, we integrated student expectations and new internet technologies to develop an online medical data mining course, titled “Practical Techniques of Medical Data Mining” (No. INSC11011), for medical students with a wide range of academic backgrounds and programming experience. Its successful application in postgraduate medical education at PUMC indicates that designing course content and online instructional methods that accommodate diversified characteristics of medical students is effective for the development of a data mining course in medical school. The diverse course content, along with representative programming examples and case studies, could meet the different expectations of targeted learners and minimize the prerequisites. In addition, the use of different instructional methods and online platforms had advantages in flexibility, which could accommodate a diverse group of medical students. The results showed that our course was able to fill the gap between student expectations and learning outcomes. In the future, we will further optimize our online course, complete the comparison of learners’ data mining skill levels before and after the class, and complete external validation.

## Appendix

Multimedia Appendix 1 Advantages and disadvantages of online platforms.

## Abbreviations

CNER: clinical named entity recognition
CRF: conditional random field
EHR: electronic health record
ICU: intensive care unit
MedHub: Medical Data Mining - R Programming Hub
PUMC: Peking Union Medical College
QR: Quick Response
TCGA: The Cancer Genome Atlas
