# Supplementary material for: Medical Data Mining Course Development in Postgraduate Medical Education: Web-Based Survey and Case Study
Source: JMIR Med Educ. 2021 Oct 1;7(4):e24027. doi: 10.2196/24027 (PMC8520135; doi:10.2196/24027)
Supplement: Multimedia Appendix 1 [file mededu_v7i4e24027_app1.docx]

Multimedia Appendix 1. Advantages and disadvantages of online platforms.

| Platform | Description | Advantages | Disadvantages |
| --- | --- | --- | --- |
| Rain Classroom | An online-offline blended teaching tool that links PowerPoint with WeChat. | 1. It allows students to review what they have learned to consolidate their knowledge points.  2. It collects, records and analyzes in-class learning data, which can help teaching evaluation.  3. It can be bound with student IDs to facilitate teaching management. | Unable to demonstrate real-time operation. |
| Tencent Meeting | A HD video conferencing tool that allows attendees to join meetings quickly on mobile phones, PCs, tablets, and webpages. | 1. It enables real-time screen sharing on PCs and mobile devices.  2. It provides flexible ways to join meetings. | Unable to replay the teaching process. |
| WeChat | A social media application for mobiles that offers text messaging, video and voice calls, and images or videos sharing. | 1. Instant messaging facilitates discussions among teachers and students.  2. It helps to share educational resources and information conveniently. | Unable to reproduce the results of codes in real time. |
| Zoom | A platform for video, voice, content sharing, and chat runs across mobile devices, desktops, telephones, and room systems. | It supports recording meetings locally. | Unstable connection. |
| DingTalk | A communication and collaboration platform that provide message, voice and video communication, workflow management and office automation functions. | 1. It enables screen and file sharing on PCs and mobile devices.  2. It supports video playback. | Additional registration is required. |
